# Rectal Administration of Carbidopa/Levodopa

**DOI:** 10.31486/toj.24.0103

**Published:** 2025

**Authors:** Andrew Guthrie, Joshua King, Ashley Casey

**Affiliations:** ^1^ Department of Internal Medicine, Ochsner Clinic Foundation, New Orleans, LA; ^2^ The University of Queensland Medical School, Ochsner Clinical School, New Orleans, LA; ^3^ Department of Pharmacy, Ochsner Clinic Foundation, New Orleans, LA

**Keywords:** *Administration–rectal*, *carbidopa–levodopa drug combination*, *Parkinson disease*

## Abstract

**Background:**

Carbidopa/levodopa is a vital medication administered to patients with Parkinson disease. In some clinical situations, oral administration of the medication is not feasible, so alternative routes for medication delivery must be explored to prevent severe complications.

**Case Report:**

A 90-year-old male with Parkinson disease presented from a nursing home with worsening confusion and agitation. Because of the patient's condition and the inability to obtain enteric access, carbidopa/levodopa was administered rectally, resulting in an observed reduction of the patient's Parkinson symptoms.

**Conclusion:**

When patients with Parkinson disease cannot take their medications orally, rectal administration of carbidopa/levodopa may be a reasonable and effective alternative route of administration.

## INTRODUCTION

Carbidopa/levodopa is a vital medication administered to patients with Parkinson disease. The symptoms of Parkinson disease—postural instability, muscle rigidity, bradykinesia, and tremors—are related to the depletion of dopamine in the corpus striatum. Levodopa is a dopamine precursor that can cross the blood-brain barrier, convert to dopamine via decarboxylation, and reduce these symptoms. Carbidopa, a decarboxylase inhibitor, reduces the peripheral conversion of levodopa to dopamine, thereby increasing the amount of dopamine delivered to the brain.^1^

Currently, only oral formulations of this medication are available. Challenges in the acute care setting that create barriers to administering this medication can expose patients to potentially serious complications, such as dopamine agonist withdrawal syndrome that can manifest as an array of symptoms, including orthostatic hypotension, agitation, muscle stiffness, and confusion.^2^

## CASE REPORT

A 90-year-old male with Parkinson disease who was taking extended-release carbidopa/levodopa 50-200 mg twice daily and an additional sustained-release carbidopa/levodopa 12.5-50 mg daily presented from a nursing home with worsening confusion and agitation. He was ill-appearing, lethargic, and disoriented. At admission, the range of motion of his upper extremities was normal and without significant rigidity. Computed tomography of the abdomen revealed a small bowel obstruction, at which point the patient's oral intake was discontinued. A nasogastric tube was placed, but the patient removed the tube during an acute episode of agitation. Multiple attempts were made to reinsert the nasogastric tube but were unsuccessful because of lack of patient cooperation and likely nasal swelling from the previous successful insertion. The lack of enteric access precluded administration of carbidopa/levodopa.

During the next 2 days of hospitalization, the patient's cognition and rigidity notably worsened, further impeding placement of a nasogastric tube. With pharmacy assistance, a carbidopa/levodopa rectal suspension was prepared by dissolving 1 crushed carbidopa/levodopa 25-100 mg immediate-release tablet in 5 mL of water to make each dose. Each dose was administered rectally via enema every 6 hours. After 10 rectal administrations, the patient demonstrated clinical improvement. Specifically, he had a notable reduction in rigidity, enabling increased active and passive range of motion in his extremities. Additionally, the patient's mentation improved, as evidenced by increased alertness and responsiveness to verbal cues.

With these clinical improvements, a nasogastric tube was safely reinserted, allowing the patient to resume standard oral medication administration. Despite this progress, because of the patient's acute and chronic life-limiting illnesses, the decision was made to transition him to comfort-focused care. The patient was discharged to his nursing home with hospice care, and he died shortly thereafter.

## DISCUSSION

Carbidopa/levodopa is the standard of care for the treatment of the primary motor symptoms of Parkinson disease. Levodopa is thought to be exclusively absorbed via sodium-dependent L-neutral amino acid channels located within the duodenum and jejunum.^3^ A limited number of studies have investigated rectal absorption of levodopa. In 1981, Eisler et al did not find an increase in serum levodopa levels or discernable clinical benefit associated with rectal tablet, suppository, or powder insufflation.^4^ Despite the findings from the Eisler et al study, anecdotal case reports have since detailed positive clinical outcomes following rectal carbidopa/levodopa administration. In 2001, Cooper et al reported the recovery of speech and spontaneous movement in a patient within 48 hours of rectal administration of a strongly acidic suspension of carbidopa/levodopa.^5^ Our institution did not have the necessary compounding ingredients or a compounding formula developed to create an acidified rectal suspension for our patient as described by Cooper et al; however, our patient still showed signs of clinical improvement with the simplified compound that we used. In 2015, Vogelzang et al described some alleviation of their patient's motor symptoms and a corresponding rise in serum levodopa level after the rectal administration of an unspecified compound of carbidopa/levodopa.^6^

While no studies have evaluated the safety of rectally administered carbidopa/levodopa, to our knowledge, no adverse events associated with rectal administration have been reported.

## CONCLUSION

Clinicians taking care of patients with Parkinson disease are often confronted with complications that prohibit the administration of oral carbidopa/levodopa. Despite the case reports that anecdotally support the rectal administration of carbidopa/levodopa, the limited scientific studies conducted in this area have failed to establish any discernible benefit. When patients with Parkinson disease cannot tolerate oral intake, the placement of a nasogastric tube for administration of carbidopa/levodopa is recommended. However, in cases where tube placement is not feasible, rectal administration of carbidopa/levodopa may be a reasonable alternative.

## Notes

*This article meets the Accreditation Council for Graduate Medical Education and the American Board of Medical Specialties Maintenance of Certification competencies for Patient Care and Medical Knowledge.*
